# Underwater 3D Doppler-Angle Target Tracking with Signal Time Delay

**DOI:** 10.3390/s20143869

**Published:** 2020-07-10

**Authors:** Jun Su, Yaan Li, Wasiq Ali, Xiaohua Li, Jing Yu

**Affiliations:** 1 School of Marine Science and Technology, Northwestern Polytechnical University, Xi’an 710072, China; kaimier2010@mail.nwpu.edu.cn (J.S.); wasiqali@mail.nwpu.edu.cn (W.A.); yujing@nwpu.edu.cn (J.Y.); 2 Shaanxi Key Laboratory for Network Computing and Security Technology, School of Computer Science and Engineering, Xi’an University of Technology, Xi’an 710048, China; lixiaohua@xaut.edu.cn

**Keywords:** Doppler-angle tracking, signal time delay, three-dimensional underwater tracking, Gauss–Helmert model

## Abstract

The traditional target tracking is a process of estimating the state of a moving target using measurement information obtained by sensors. However, underwater passive acoustic target tracking will confront further challenges, among which the system incomplete observability and time delay caused by the signal propagation create a great impact on tracking performance. Passive acoustic sensors cannot accurately obtain the target range information. The introduction of Doppler frequency measurement can improve the system observability performance; signal time delay cannot be ignored in underwater environments. It varies with time, which has a continuous negative impact on the tracking accuracy. In this paper, the Gauss–Helmert model is introduced to solve this problem by expanding the unknown signal emission time as an unknown variable to the state. This model allows the existence of the previous state and current state at the same time, while handling the implicit equations. To improve the algorithm accuracy, this paper further takes advantage of the estimated state and covariance for the second stage iteration and propose the Gauss–Helmert iterated Unscented Kalman filter under a three-dimensional environment. The simulation shows that the proposed method in this paper shows superior estimation accuracy and more stable performance compared with other filtering algorithms in underwater environments.

## 1. Introduction

In recent decades, multiple new techniques have been applied to underwater tracking systems in the literature. Among various underwater tracking methods, the passive target tracking methods have attracted great interest [1,2,3,4,5] by the research community. Compared to the active system, the costs of operation and maintenance for passive systems are lower [6]. In some special applications, a passive system has the advantage of keeping covert and hardly being detected [7]. The angle-only tracking and Doppler-angle tracking problems relied on acoustic sensors and belonged to the passive target tracking [8,9]. The main problem is estimating the unknown target state through the noisy measurements acquired by acoustic sensors [10,11].

The high nonlinearity of the measurement system makes the Doppler-angle tracking problem complex and difficult to solve [12,13]. Various nonlinear filtering algorithms for Doppler-angle tracking can be roughly divided into two types. One approach is the pseudo linearization method, which has less calculation with a wide range of application compared to other methods [14,15,16]. However, the result of the estimation is biased, which will continuously affect the tracking process even if the number of measurements increased. Another one is the recursive nonlinear filter method, including the extended Kalman filter (EKF) and unscented Kalman filter (UKF) based methods. The basic idea of the former method is to utilize the first-order or second-order Taylor series expansion, which is easy to implement in various problems [17,18]. However, the disadvantage of linearization is that it will greatly increase the filtering error when handling highly nonlinear systems, which may eventually lead to divergence of the tracking system. The latter method is to fit the probability density distribution of the nonlinear equations with fixed parameters through unscented transform [19,20,21]. UKF avoids the loss of higher-order terms in Taylor series expansion caused by linearization and improves accuracy [22,23].

The frequency measurements can provide extra target motion information, which makes the Doppler-angle tracking better than angle-only tracking in observability and estimation performance [24,25]. The condition is that the target’s radiation frequency must remain constant during the tracking period [26]. In this case, as long as the system satisfies the observability condition for a single observer, even the observers are not required to maneuver, which is very useful in practical applications [27].

Passive target tracking often relies on acoustic sensors to obtain the angle measurements, and acoustic signals cannot be immediately propagated from the target to the acoustic sensors. However, the time delay caused by a signal spread in the medium is usually considered by having little effect on filtering results in the general Doppler-angle tracking problem [28]. In other words, the signal is often regarded as being transmitted from the target to the sensors instantly. This assumption is valid when the signal speed is much greater than the target motion speed. For example, in the radar systems, the propagation speed of radio waves is approximately equal to 3×8 m/s. In this case, the signal propagation delay is generally negligible. However, this assumption is too ideal in the underwater environment. As the only reliable signal that can propagate long distances in an underwater environment, the underwater acoustic speed is about 1480 m/s, which is much less than the propagation speed of light or radio. Under this circumstance, the impact of signal propagation delay is more apparent. The position of the target continuously varies while the signal travels to the observer [29]. As a consequence, the estimated target state and its true value will be remarkably different if the time delay is neglected [30].

The main issues that make the passive target tracking with signal propagation a challenging problem are time varying delay and the incomplete observability of the system [31,32,33]. In order to get the target state, multiple strategies have been applied in this domain. In early researches, the interval between two consecutive signals is regarded as an unknown parameter [34,35], which is calculated recursively through a linear search method. This method has two disadvantages, for example, the signal transmission time interval is calculated directly during the iteration process without considering noise while iterative calculation may be excessive due to the linear search method. Another strategy extends the state, and the signal emission time becomes one of the variables [36,37,38]. Unlike the traditional Gauss–Markov model (GMM), the Gauss–Hermit model (GHM) in these algorithms can be used to describe strongly complex state equations, which allows the existence of the previous state and the current state at the same time. Compared with the first method, the GHM method equipped with Unscented transformation has better estimation performance. However, the application of these methods are relatively limited. In [38], the method is mainly aimed at eliminating the unknown time offset between two stationary observers under the influence of time propagation delay. The approach in [36] utilizes a maneuvering single observation station to deal with the bearing-only target tracking. Due to the lack of special optimization, the performance improvement of this method for underwater targets is not significant, and it cannot be directly applied in 3D space. Meanwhile, the approach has been extended to the Doppler-bearing tracking in [37], which rely on an electronic support measures/electro-optical (ESM/EO) sensor. However, it cannot be deployed in an underwater environment.

In this paper, we investigate the Doppler-angle tracking with the propagation delay by the single maneuvering observer in an underwater environment, and expand the application to the three-dimensional space. A novel method is proposed to deal with the underwater Doppler-angle tracking problem.

The remainder of the paper is planned as the following five sections. Section 2 investigates the Doppler-angle target tracking problem with the system state and measurement model in an underwater three-dimension environment. In Section 3, the problems caused by signal time delay is investigated. The new Gauss–Helmert Iterated Unscented Kalman Filter (GH-IUKF) algorithm is derived in Section 4. Section 5 provides a comparative analysis of the performance for the proposed algorithm and other different algorithms under various conditions. Finally, the conclusion is drawn in Section 6.

## 2. Problem Description

### 2.1. System Model Description

Assuming the target is moving at a constant velocity in an underwater environment. We deploy a single maneuvering observer, which includes three passive sensors together. The location of the observer is considered to be known in advance. These sensors are used for receiving the bearing information, elevation information and frequencies of the target. Define $x^{T}=\left(x^{T},y^{T},z^{T}\right)$ as the location and ${\dot{x}}^{T}=\left({\dot{x}}^{T},{\dot{y}}^{T},{\dot{z}}^{T}\right)$ as the velocity of the target, *f* is the Doppler frequency from the target while $t_{k}$ is the time of $k^{th}$ observer sampling. Then the target state vector can be expressed as

(1) $$x^{T}\left(t_{k}\right)={\left[x^{T}\left(t_{k}\right)\;\;y^{T}\left(t_{k}\right)\;\;z^{T}\left(t_{k}\right)\;\;{\dot{x}}^{T}\left(t_{k}\right)\;\;{\dot{y}}^{T}\left(t_{k}\right)\;\;{\dot{z}}^{T}\left(t_{k}\right)\;\;f^{T}\left(t_{k}\right)\right]}^{\prime },$$

Similarly, the observer motion state is defined as

(2) $$x^{S}\left(t_{k}\right)={\left[x^{S}\left(t_{k}\right)\;\;y^{S}\left(t_{k}\right)\;\;z^{S}\left(t_{k}\right)\;\;{\dot{x}}^{S}\left(t_{k}\right)\;\;{\dot{y}}^{S}\left(t_{k}\right)\;\;{\dot{z}}^{S}\left(t_{k}\right)\right]}^{\prime }.$$

The relationship of the target state between two consecutive times $t_{k}$ and $t_{k-1}$ can be described by the discrete state transition model as

(3) $$x^{T}\left(t_{k}\right)=F\left(\;t_{k}\left|t_{k-1}\right.\right)x^{T}\left(t_{k-1}\right)+\Gamma \left(\;t_{k}\left|t_{k-1}\right.\right)w\left(\;t_{k}\left|t_{k-1}\right.\right),$$

where $F(\;t_{k}|t_{k-1})$ denotes the state transition matrix and $w(\;t_{k}|t_{k-1})$ denotes the zero-mean Gaussian white noise, $\Gamma (\;t_{k}|t_{k-1})$ is the system noise-driven noise matrix. For the constant velocity (CV) model

(4) $$F\left(\;t_{k}\left|t_{k-1}\right.\right)=\left[\begin{array}{ccccccc} 1 & 0 & 0 & T & 0 & 0 & 0\\ 0 & 1 & 0 & 0 & T & 0 & 0\\ 0 & 0 & 1 & 0 & 0 & T & 0\\ 0 & 0 & 0 & 1 & 0 & 0 & 0\\ 0 & 0 & 0 & 0 & 1 & 0 & 0\\ 0 & 0 & 0 & 0 & 0 & 1 & 0\\ 0 & 0 & 0 & 0 & 0 & 0 & 1 \end{array}\right]$$

(5) $$\Gamma =\left[\begin{array}{cccc} T^{2}/2 & 0 & 0 & 0\\ 0 & T^{2}/2 & 0 & 0\\ 0 & 0 & T^{2}/2 & 0\\ T & 0 & 0 & 0\\ 0 & T & 0 & 0\\ 0 & 0 & T & 0\\ 0 & 0 & 0 & 1 \end{array}\right]$$

where *T* is the observer sampling interval. The measurement equation can be written as

(6) $$z\left(t_{k}\right)=h\left(x\left(t_{k}\right)\right)+v\left(t_{k}\right).$$

where $h(x\left(t_{k}\right))$=${\left[\theta \left(t_{k}\right)\;\varphi \left(t_{k}\right)\;f^{S}\left(t_{k}\right)\right]}^{\prime }$ denotes nonlinear measurement function, $v(t_{k})$ is the measurement noise.

### 2.2. Doppler-Angle Tracking in Three-Dimension Space

In this section, we analyze the geometric relationship between the target and the observer, then apply the frequency information to the three-dimensional space.

**Figure 1:**
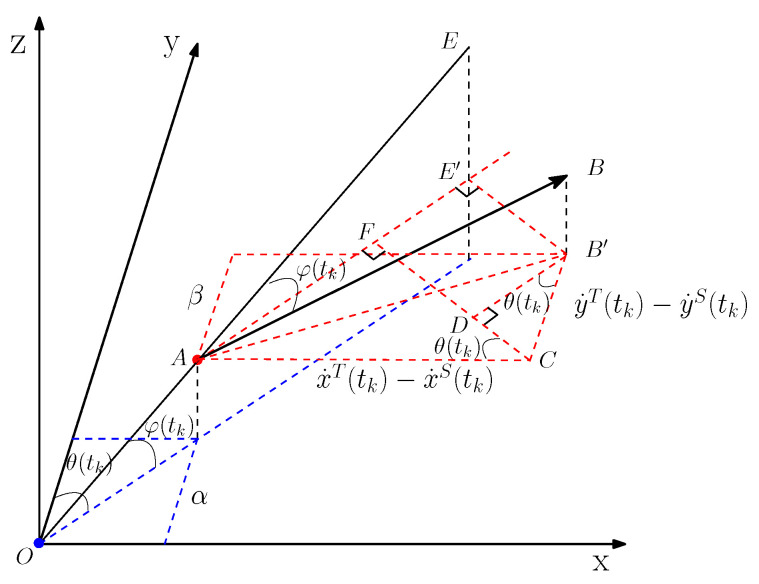
Geometry for Doppler-angle tracking in a three-dimension environment.

As shown in Figure 1, the target and observer are located at points *A* and *O* respectively. The target is moving from point *A* towards *B* at the instant $t_{k}$. α denotes the XOY plane, β denotes the plane parallel to the XOY plane and passing through point *A*. θ represents the horizontal bearing angle and φ denotes the vertical pitch angle.

Assuming that $\overset{⟶}{AB}$ represents the instantaneous velocity of the target, $B^{\prime }$ is the projection of point *B* in the β plane, then $\overset{⟶}{AC}={\dot{x}}^{T}\left(t_{k}\right)-{\dot{x}}^{S}\left(t_{k}\right),\overset{⟶}{CB^{\prime }}={\dot{y}}^{T}\left(t_{k}\right)-{\dot{y}}^{S}\left(t_{k}\right)$. According to the trigonometry, the relative radial velocity $V_{XOY}^{r}$ in the β plane can be expressed as

(7) $$V_{XOY}^{r}\left(t_{k}\right)=\overset{⟶}{AF}+\overset{⟶}{DB^{\prime }}=\left({\dot{x}}^{T}\left(t_{k}\right)-{\dot{x}}^{S}\left(t_{k}\right)\right)\sin\theta \left(t_{k}\right)+\left({\dot{y}}^{T}\left(t_{k}\right)-{\dot{y}}^{S}\left(t_{k}\right)\right)\cos\theta \left(t_{k}\right),$$

Then the relative radial velocity $V^{r}$ in three-dimension space is calculated by

(8) $$V^{r}\left(t_{k}\right)=V_{XOY}^{r}\left(t_{k}\right)/\cos\varphi \left(t_{k}\right)=\frac{\left({\dot{x}}^{T}\left(t_{k}\right)-{\dot{x}}^{S}\left(t_{k}\right)\right)\sin\theta \left(t_{k}\right)+\left({\dot{y}}^{T}\left(t_{k}\right)-{\dot{y}}^{S}\left(t_{k}\right)\right)\cos\theta \left(t_{k}\right)}{\cos\varphi (t_{k})},$$

where

(9) $$\sin\theta \left(t_{k}\right)=\frac{x^{T}\left(t_{k}\right)-x^{S}\left(t_{k}\right)}{l(t_{k})}$$

(10) $$\cos\theta \left(t_{k}\right)=\frac{x^{T}\left(t_{k}\right)-x^{S}\left(t_{k}\right)}{l(t_{k})}$$

(11) $$\cos\varphi \left(t_{k}\right)=\frac{l(t_{k})}{d(t_{k})}.$$

where $l\left(t_{k}\right)=\sqrt{{\left(x^{T}\left(t_{k}\right)-x^{S}\left(t_{k}\right)\right)}^{2}+{\left(y^{T}\left(t_{k}\right)-y^{S}\left(t_{k}\right)\right)}^{2}}$ represents the horizontal distance between the target and observer. Similarly, $d\left(t_{k}\right)=\sqrt{{\left(x^{T}\left(t_{k}\right)-x^{S}\left(t_{k}\right)\right)}^{2}+{\left(y^{T}\left(t_{k}\right)-y^{S}\left(t_{k}\right)\right)}^{2}+{\left(z^{T}\left(t_{k}\right)-z^{S}\left(t_{k}\right)\right)}^{2}}$ denotes the distance between the target and observer.

When the target moves as a certain radial velocity relative to the observer, the frequency of the target signal will be affected by the Doppler shift during the propagation, then the frequency measurements $f^{S}$ obtained by the observer without noise is given by

(12) $$f^{S}\left(t_{k}\right)=f^{T}\left(t_{k}\right)\left(1+\frac{V^{r}\left(t_{k}\right)}{c}\right),$$

where c is the underwater sound speed. Substituting Equations (7)–(10) into (11), the frequency measurements without noise can be expressed as

(13) $$\begin{array}{cc} f^{S}\left(t_{k}\right)= & f^{T}\left(t_{k}\right)\left(1+\frac{d\left(t_{k}\right)\left[\left({\dot{x}}^{T}\left(t_{k}\right)-{\dot{x}}^{S}\left(t_{k}\right)\right)\left(x^{T}\left(t_{k}\right)-x^{S}\left(t_{k}\right)\right)+\left({\dot{y}}^{T}\left(t_{k}\right)-{\dot{y}}^{S}\left(t_{k}\right)\right)\left(y^{T}\left(t_{k}\right)-y^{S}\left(t_{k}\right)\right)\right]}{[{\left(x^{T}\left(t_{k}\right)-x^{S}\left(t_{k}\right)\right)}^{2}+{\left(y^{T}\left(t_{k}\right)-y^{S}\left(t_{k}\right)\right)}^{2}]c}\right), \end{array}$$

Similarly, the angle measurement functions can be expressed as

(14) $$\begin{array}{cc} \theta (t_{k}) & =\arctan(\frac{x^{T}\left(t_{k}\right)-x^{S}\left(t_{k}\right)}{y^{T}\left(t_{k}\right)-y^{S}\left(t_{k}\right)}) \end{array}$$

(15) $$\begin{array}{cc} \varphi (t_{k}) & =\arctan(\frac{z^{T}\left(t_{k}\right)-z^{S}\left(t_{k}\right)}{l(t_{k})}). \end{array}$$

Then the measurements for the Doppler-angle only system satisfy the following equations as

(16) $$\begin{array}{cc} z_{\theta }\left(t_{k}\right) & =h_{1}\left(x\left(t_{k}\right)\right)+v_{\theta }(t_{k})=\arctan\left(\frac{x^{T}\left(t_{k}\right)-x^{S}\left(t_{k}\right)}{y^{T}\left(t_{k}\right)-y^{S}\left(t_{k}\right)}\right)+v_{\theta }(t_{k}) \end{array}$$

(17) $$\begin{array}{cc} z_{\varphi }\left(t_{k}\right) & =h_{2}\left(x\left(t_{k}\right)\right)+v_{\varphi }(t_{k})=\arctan\left(\frac{z^{T}\left(t_{k}\right)-z^{S}\left(t_{k}\right)}{l(t_{k})}\right)+v_{\varphi }(t_{k}) \end{array}$$

(18) $$\begin{array}{cc} z_{f}\left(t_{k}\right) & =h_{3}\left(x\left(t_{k}\right)\right)+v_{f}(t_{k})=f^{T}\left(t_{k}\right)\left(1+\frac{V^{r}\left(t_{k}\right)}{c}\right)+v_{f}(t_{k}). \end{array}$$

where $v_{\theta }\left(t_{k}\right)$, $v_{\varphi }\left(t_{k}\right)$, and $v_{f}\left(t_{k}\right)$ are the random measurement errors, which follows the zero-mean Gaussian distribution respectively.

## 3. Signal Propagation Delay Analysis

In this section, we analyzed the effect of unknown signal propagation delay on the tracking system and introduced a nonlinear Gauss–Helmert model to solve this problem.

**Figure 2:**
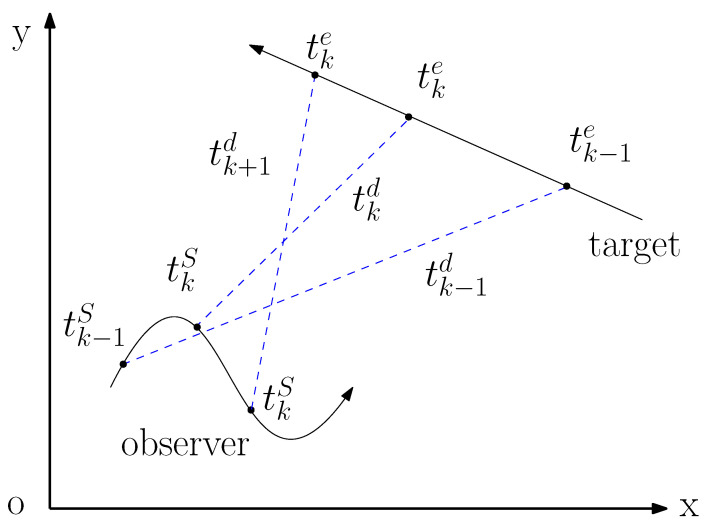
Tracking Scenario in the XOY plane.

In Figure 2, $t_{k}^{e}$ denotes the signal emission time within one interval of sampling. $t_{k}^{S}$ is the time when the sensor receives signal and $t_{k}^{d}$ represents the signal time delay. The signal emission time is given by

(19) $$t_{k}^{e}=t_{k}^{S}-t_{k}^{d}$$

The relationships among $t_{k}^{S}$ and $t_{k}^{e}$ are given by

(20) $$\begin{array}{cc} \; & t_{k}^{e}-t_{k-1}^{e}=\Delta_{k}^{e} \end{array}$$

(21) $$\begin{array}{cc} \; & t_{k}^{S}-t_{k-1}^{S}=T \end{array}$$

(22) $$\begin{array}{cc} \; & T=\Delta_{k}^{e}+\tau . \end{array}$$

where $\Delta_{k}^{e}$ is the interval between two signal emission time, *T* is the interval of sampling, and τ is a varying time offset.

**Figure 3:**
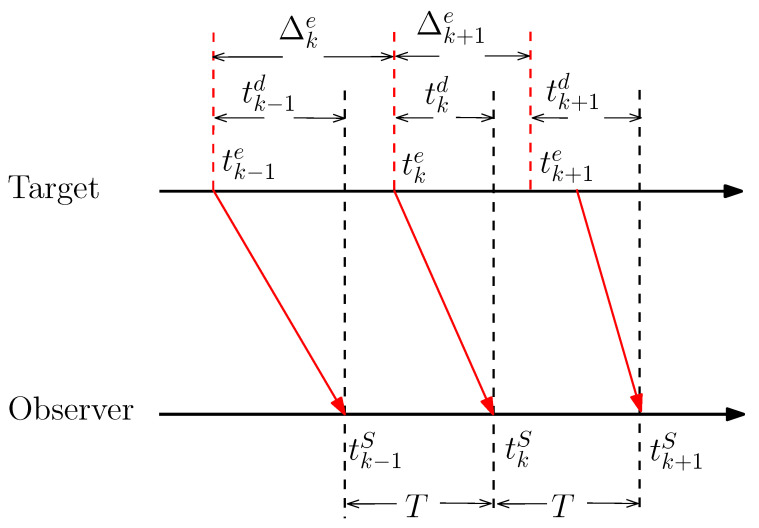
The illustration of time series.

According to the description of Figure 3, the time sequence of the tracking system under signal time delay is distinct from the one without delay. The signal transmission delay will have a strong impact on the system, which leads to $\Delta_{k}^{e}$ being not equal to the sensor interval. As the result, $\Delta_{k}^{e}$ will be a time varying parameter according to the target state. In Section 2, the discrete dynamic model requires that time intervals between the adjacent state vectors should always be the same. When the signal propagation delay cannot be ignored, the discrete dynamic Equation (3) is not suitable for this situation.

We regard $t_{k}^{e}$ as an unknown variable in the state vector. However, two adjacent state vectors $x^{T}\left(t_{k}^{e}\right)$ and $x^{T}\left(t_{k-1}^{e}\right)$ will exist in the state transition function simultaneously, which cannot be solved by the nonlinear GMM.

Here, we introduce the implicit equations based on GHM to handle this problem as

(23) $$s\left[x^{*}\left(t_{k}^{e}\right),x^{*}\left(t_{k-1}^{e}\right)\right]+\Gamma \left(\;t_{k}^{e}\left|t_{k-1}^{e}\right.\right)w\left(t_{k-1}^{e}\right)=0_{n}.$$

where $0_{n}$ represents the (n)×(n) zero vector, and n is the state dimension. $w(t_{k-1}^{e})$ is the noise which follows the Gaussian distribution, $\Gamma (\;t_{k}^{e}\left|t_{k-1}^{e}\right.)$ is the system noise gain matrix. The state vector $x^{T}\left(t_{k}^{e}\right)$ in (1) is extended as

(24) $$x^{*}\left(t_{k}^{e}\right)={\left[x^{T}\left(t_{k}^{e}\right)\;y^{T}\left(t_{k}^{e}\right)\;z^{T}\left(t_{k}^{e}\right)\;{\dot{x}}^{T}\left(t_{k}^{e}\right)\;{\dot{y}}^{T}\left(t_{k}^{e}\right)\;{\dot{z}}^{T}\left(t_{k}^{e}\right)\;f_{k}^{T}\;t_{k}^{e}\right]}^{\prime }.$$

The specific form of the implicit state equations under the CV model are given by

(25) $$s\left(\;\cdot \;\right)={\left[s_{1}\left(\;\cdot \;\right)\;s_{2}\left(\;\cdot \;\right)\;s_{3}\left(\;\cdot \;\right)\;s_{4}\left(\;\cdot \;\right)\;s_{5}\left(\;\cdot \;\right)\;s_{6}\left(\;\cdot \;\right)\;s_{7}\left(\;\cdot \;\right)\;s_{8}\left(\;\cdot \;\right)\right]}^{\prime },$$

where

(26) $$\begin{array}{cc} s_{1} & =x^{T}\left(t_{k}^{e}\right)\;-x^{T}\left(t_{k-1}^{e}\right)-{\dot{x}}^{T}\left(t_{k-1}^{e}\right)\Delta_{k}^{e}\;\; \end{array}$$

(27) $$\begin{array}{cc} s_{2} & =y^{T}\left(t_{k}^{e}\right)\;-y^{T}\left(t_{k-1}^{e}\right)-{\dot{y}}^{T}\left(t_{k-1}^{e}\right)\Delta_{k}^{e} \end{array}$$

(28) $$\begin{array}{cc} s_{3} & =z^{T}\left(t_{k}^{e}\right)\;-z^{T}\left(t_{k-1}^{e}\right)-{\dot{z}}^{T}\left(t_{k-1}^{e}\right)\Delta_{k}^{e} \end{array}$$

(29) $$\begin{array}{cc} s_{4} & ={\dot{x}}^{T}\left(t_{k}^{e}\right)\;-{\dot{x}}^{T}\left(t_{k-1}^{e}\right) \end{array}$$

(30) $$\begin{array}{cc} s_{5} & ={\dot{y}}^{T}\left(t_{k}^{e}\right)\;-{\dot{y}}^{T}\left(t_{k-1}^{e}\right) \end{array}$$

(31) $$\begin{array}{cc} s_{6} & ={\dot{z}}^{T}\left(t_{k}^{e}\right)\;-{\dot{z}}^{T}\left(t_{k-1}^{e}\right) \end{array}$$

(32) $$\begin{array}{cc} s_{7} & =f^{T}\left(t_{k}^{e}\right)-f^{T}\left(t_{k-1}^{e}\right) \end{array}$$

(33) $$\begin{array}{cc} s_{8} & =t_{k}^{e}+t_{k}^{d}-t_{k}^{s} \end{array}$$

in addition

(34) $$t_{k}^{d}\;=d\left(t_{k}^{e}\right)/c$$

(35) $$d\left(t_{k}^{e}\right)=\sqrt{{\left(x^{T}\left(t_{k}^{e}\right)-x^{S}\left(t_{k}^{S}\right)\right)}^{2}+{\left(y^{T}\left(t_{k}^{e}\right)-y^{S}\left(t_{k}^{S}\right)\right)}^{2}+{\left(z^{T}\left(t_{k}^{e}\right)-z^{S}\left(t_{k}^{S}\right)\right)}^{2}}.$$

where $d(t_{k}^{e})$ denotes the distance from the target to the observer and *c* is the constant underwater sound speed. Functions $s_{1}$ to $s_{6}$ are the constraint functions for the CV model, which denotes the state transition of target motion parameters between two neighbor instants $t_{k}^{e}$ and $t_{k-1}^{e}$. $s_{7}$ represents the signal frequency which is varying by the Doppler effect. $s_{8}$ is the time delay constraint, which describes the relationship for the time series.

Affected by signal propagation delay, the state transition matrix for the CV model is rewritten as

(36) $$F\left(\;t_{k}^{e}\left|t_{k-1}^{e}\right.\right)=\left[\begin{array}{cccccccc} 1 & 0 & 0 & \Delta_{k}^{e} & 0 & 0 & 0 & 0\\ 0 & 1 & 0 & 0 & \Delta_{k}^{e} & 0 & 0 & 0\\ 0 & 0 & 1 & 0 & 0 & \Delta_{k}^{e} & 0 & 0\\ 0 & 0 & 0 & 1 & 0 & 0 & 0 & 0\\ 0 & 0 & 0 & 0 & 1 & 0 & 0 & 0\\ 0 & 0 & 0 & 0 & 0 & 1 & 0 & 0\\ 0 & 0 & 0 & 0 & 0 & 0 & 1 & 0\\ 0 & 0 & 0 & 0 & 0 & 0 & 0 & 1 \end{array}\right],$$

To describe the uncertainty of the variable, the process noise gain matrix and the covariance of $w(t_{k-1}^{e})$ are given by

(37) $$\Gamma \left(\;t_{k}^{e}\left|t_{k-1}^{e}\right.\right)=\left[\begin{array}{ccccc} \Delta_{k}^{e^{2}/2} & 0 & 0 & 0 & 0\\ 0 & \Delta_{k}^{e^{2}/2} & 0 & 0 & 0\\ 0 & 0 & \Delta_{k}^{c^{2}/2} & 0 & 0\\ \Delta_{k}^{e} & 0 & 0 & 0 & 0\\ 0 & \Delta_{k}^{c} & 0 & 0 & 0\\ 0 & 0 & \Delta_{k}^{c} & 0 & 0\\ 0 & 0 & 0 & 1 & 0\\ 0 & 0 & 0 & 0 & 1 \end{array}\right],$$

(38) $$q=diag(\sigma_{\ddot{x}}^{2}\;\sigma_{\ddot{y}}^{2}\;\sigma_{\ddot{z}}^{2}\;\sigma_{f}^{2}\;\sigma_{te}^{2}),$$

where $\sigma_{\ddot{x}}$, $\sigma_{\ddot{y}}$ and $\sigma_{\ddot{z}}$ are the noise standard deviations to describe the unknown small acceleration for the *x*, *y* and *z* axis, respectively. $\sigma_{f}$ and $\sigma_{te}$ are the system process noise standard deviations to show the random disturbance of the system.

The system process noise matrix is

(39) $$Q\left(\;t_{k}^{e}\left|t_{k-1}^{e}\right.\right)=\Gamma \left(\;t_{k}^{e}\left|t_{k-1}^{e}\right.\right)q\Gamma \left(\;t_{k}^{e}\left|t_{k-1}^{e}\right.\right).$$

Note that the geometric relationship in the measurement Equations (16)–(18) will not change here, however, the measurements received by the observer have a different time axis from the target state. The measurement equations for the Doppler-angle only system with signal propagation delay are given by

(40) $$\begin{array}{cc} z_{\theta }\left(t_{k}^{S}\right) & =h_{1}\left(x\left(t_{k}^{*}\right)\right)+v_{\theta }(t_{k}^{S}) \end{array}$$

(41) $$\begin{array}{cc} z_{\varphi }\left(t_{k}^{S}\right) & =h_{2}\left(x\left(t_{k}^{*}\right)\right)+v_{\varphi }(t_{k}^{S}) \end{array}$$

(42) $$\begin{array}{cc} z_{f}\left(t_{k}^{S}\right) & =h_{3}\left(x\left(t_{k}^{*}\right)\right)+v_{f}(t_{k}^{S}). \end{array}$$

## 4. Gauss–Helmert Iterated Unscented Kalman Filter

The traditional nonlinear filtering is mainly based on the EKF, which has a wide range of applications and low calculation cost. However, it also has the disadvantages of low accuracy and poor stability, and is only suitable for the weak nonlinear Gaussian environments. Particle filtering can be used for nonlinear non-Gaussian systems, however, the main problem is that a great number of samples are required to approximate the system posterior probability density. The complexity of the algorithm is positively related to the number of samples which describes the posterior probability distribution. UKF combines the unscented transformation and Kalman filter. UKF approximates the n-dimensional target sampling points by designing the weighted point σ, and calculates these σ points propagated by the nonlinear function. Then, the updated state by the nonlinear equation is obtained. Different from the EKF filter, the state processed by UKF reserves more nonlinear characteristics and keeps better estimation performance. For the non-Gaussian measurements, the estimation accuracy of UKF approximates to at least the second-order of the Taylor series expansion. For the non-Gaussian noise, the estimation accuracy of UKF approximates at least the second-order of the Taylor series expansion. The UKF can further approximate the third order of Taylor series expansion for all nonlinear equations with Gaussian noise [21]. Moreover, the computational complexity of UKF is much smaller than that of PF. Therefore, we choose the UKF as the main framework of the filter to deal with the Doppler-angle tracking problem with signal time delay.

In the previous section, under the influence of the signal propagation delay, the unknown parameters of the target state, the state transition equations and the measurement equations are different from the usual Doppler-angle tracking. Similarly, the Iterated Unscented Kalman filter (IUKF) implementation process also needs to make a response adjustment. The estimation of $x^{*}\left(t_{k}^{e}\right)$ can be acquired from the following steps.

1. Select the sigma sample points

For a given state $x^{*}\left(t_{k-1}^{e}\right)$ and corresponding error covariance $P(t_{k-1}^{e})$ at the instant $t_{k-1}^{e}$, the sigma points matrix $\xi (t_{k-1}^{e})$ with associated weights $w^{i}$ are calculated as

(43) $$\begin{array}{cc} \xi (t_{k-1}^{e}) & =[{\hat{x}}^{*}\left(t_{k-1}^{e}\right){\hat{x}}^{*}\left(t_{k-1}^{e}\right)+\sqrt{\left(n+\lambda \right)P\left(t_{k-1}^{e}\right)}{\hat{x}}^{*}\left(t_{k-1}^{e}\right)-\sqrt{\left(n+\lambda \right)P\left(t_{k-1}^{e}\right)}], \end{array}$$

(44) $$\begin{array}{cc} w_{0}^{m} & =\frac{\lambda }{n+\lambda }, \end{array}$$

(45) $$\begin{array}{cc} w_{0}^{c} & =\frac{\lambda }{n+\lambda }+\left(1-\alpha^{2}+\beta \right), \end{array}$$

(46) $$\begin{array}{cc} w_{i}^{m} & =w_{i}^{c}=\frac{\lambda }{2(n+\lambda )},i=1,2,\cdots ,2n. \end{array}$$

where *n* denotes the state dimension. $\lambda =\alpha^{2}\left(n+\kappa \right)-n$ is a scaling parameter which can affect the estimation accuracy. α and κ are the parameters to control the spread of the sigma points. β is determined by the state vector distribution.

2. The predicted state

According to (23), the function group s(·) contains state vectors at the instant $t_{k-1}^{e}$ and $t_{k}^{e}$ simultaneously. The sigma points cannot be propagated by these implicit functions. The solution of the above equations can be equivalently treated as an unconstrained optimization problem. In numerical optimization, iterative methods are generally used to solve such problems. In this paper, we adopt the Gauss–Newton method to calculate the local optimum solution. For the each predicted sigma point vector $\xi_{i}\left(t_{k}^{e}|t_{k-1}^{e}\right)$$(\xi_{i}$ represent the (i+1)th column of the matrix ξ.i=0,1,2,⋯,2n), the iterative process is given by

(47) $$\begin{array}{cc} {\left[\xi_{i}\left(t_{k}^{e}|t_{k-1}^{e}\right)\right]}^{l} & ={\left[\xi_{i}\left(t_{k}^{e}|t_{k-1}^{e}\right)\right]}^{l-1}-{\left(J_{s}^{T}J_{s}\right)}^{-1}J_{s}^{T}s\left({\left[\xi_{i}\left(t_{k}^{e}|t_{k-1}^{e}\right)\right]}^{l-1},\xi_{i}\left(t_{k-1}^{e}\right)\right), \end{array}$$

where *l* is the iteration index, $J_{s}$ is the Jacobian matrix given by

(48) $$\begin{array}{cc} J_{s} & =\frac{\partial \;s\left({\left[\xi_{i}\left(t_{k}^{e}|t_{k-1}^{e}\right)\right]}^{l-1},\xi^{i}(t_{k-1}^{e})\right)}{\partial {\left[\xi_{i}\left(t_{k}^{e}|t_{k-1}^{e}\right)\right]}^{l-1}}\\ \; & =\left[\begin{array}{cccccccc} 1 & 0 & 0 & 0 & 0 & 0 & 0 & -{\dot{x}}^{T}\left(t_{k-1}^{e}\right)\\ 0 & 1 & 0 & 0 & 0 & 0 & 0 & -{\dot{y}}^{T}\left(t_{k-1}^{e}\right)\\ 0 & 0 & 1 & 0 & 0 & 0 & 0 & -{\dot{z}}^{T}\left(t_{k-1}^{e}\right)\\ 0 & 0 & 0 & 1 & 0 & 0 & 0 & 0\\ 0 & 0 & 0 & 0 & 1 & 0 & 0 & 0\\ 0 & 0 & 0 & 0 & 0 & 1 & 0 & 0\\ 0 & 0 & 0 & 0 & 0 & 0 & 1 & 0\\ \frac{x^{T}-x^{S}}{dc} & \frac{y^{T}-y^{S}}{dc} & \frac{z^{T}-z^{S}}{dc} & 0 & 0 & 0 & 0 & 1 \end{array}\right]. \end{array}$$

For simplicity, the time label at the last row of the matrix in Equation (48) is omitted. Define the sum of squared residuals for the Gauss–Newton method as

(49) $$SSR^{l}=\sum_{i=0}^{n}{\left[0-s\left({\left[\xi_{i}\left(t_{k}^{e}|t_{k-1}^{e}\right)\right]}^{l-1},\xi_{i}\left(t_{k-1}^{e}\right)\right)\right]}^{2},$$

For the given iteration convergence threshold parameter *a*, the accuracy criterion for iteration is denoted as

(50) $$\left|\frac{SSR^{l}-SSR^{l-1}}{SSR^{l}}\right|<a.$$

The the initial value for iteration process can be calculated according to [36], or it can be approximately replaced by

(51) $$[\xi_{i}(t_{k}^{e}|t_{k-1}^{e}{)]}^{0}={\hat{\xi }}_{i}(t_{k}^{e}\left|t_{k-1}^{e}\right),$$

The convergence criteria of the Gauss–Newton method is given in Appendix A.

The prediction state and corresponding error covariance are given by

(52) $$\begin{array}{c} \hat{x}(t_{k}^{e}\left|t_{k-1}^{e}\right)=\sum_{i=0}^{2n}w_{i}^{m}{\hat{\xi }}_{i}(t_{k}^{e}\left|t_{k-1}^{e}\right) \end{array}$$

(53) $${\tilde{x}}_{i}^{\xi }\left(t_{k}^{e}|t_{k-1}^{e}\right)={\hat{\xi }}_{i}(t_{k}^{e}|t_{k-1}^{e})-\hat{x}(t_{k}^{e}\left|t_{k-1}^{e}\right)$$

(54) $$P(t_{k}^{e}|t_{k-1}^{e})\approx \sum_{i=0}^{2n}w_{i}^{c}{\tilde{x}}_{i}^{\xi }(t_{k}^{e}|t_{k-1}^{e}){\left({\tilde{x}}_{i}^{\xi }(t_{k}^{e}|t_{k-1}^{e})\right)}^{\prime }+Q(t_{k}^{e}\left|t_{k-1}^{e}\right).$$

where $Q(t_{k}^{e}\left|t_{k-1}^{e}\right)$ is correlates with the state, but this dependence is neglectable during the calculation process.

3. Measurement update

For the prediction state $\hat{x}(t_{k}^{e}\left|t_{k-1}^{e}\right)$ and covariance $P(t_{k}^{e}|t_{k-1}^{e})$ in step 2, re-propagate the sigma points according to

(55) $$\zeta (t_{k}^{e}|t_{k-1}^{e})=[\hat{x}(t_{k}^{e}|t_{k-1}^{e})\;\;\;\;\;\;\hat{x}(t_{k}^{e}|t_{k-1}^{e})+\sqrt{\left(n+\lambda \right)P(t_{k}^{e}|t_{k-1}^{e})}\;\;\;\;\;\;\hat{x}(t_{k}^{e}\left|t_{k-1}^{e})-\sqrt{\left(n+\lambda \right)P(t_{k}^{e}|t_{k-1}^{e})}\right],$$

The one-step measurement prediction is propagated by the following measurement equations

(56) $$\begin{array}{cc} \; & {\hat{z}}_{i}(t_{k}^{S}|t_{k-1}^{S})=h\left(\zeta_{i}\right),\;\;i=0,1,\cdots ,2n \end{array}$$

(57) $$\begin{array}{cc} \; & \hat{z}(t_{k}^{S}|t_{k-1}^{S})=\sum_{i=0}^{2n}w_{i}^{m}\;{\hat{z}}_{i}(t_{k}^{S}|t_{k-1}^{S}). \end{array}$$

where $\zeta_{i}$ represent the (i+1)th column of the matrix ζ.

The state and covariance for the first stage of GH-IUKF are given by

(58) $$\begin{array}{cc} \; & {\tilde{x}}_{i}^{\zeta }\left(t_{k}^{e}|t_{k-1}^{e}\right)={\hat{\zeta }}_{i}(t_{k}^{e}|t_{k-1}^{e})-\hat{x}(t_{k}^{e}\left|t_{k-1}^{e}\right) \end{array}$$

(59) $$\begin{array}{cc} \; & {\tilde{z}}_{i}\left(t_{k}^{S}|t_{k-1}^{S}\right)={\hat{z}}_{i}(t_{k}^{S}|t_{k-1}^{S})-\hat{z}(t_{k}^{S}|t_{k-1}^{S}) \end{array}$$

(60) $$\begin{array}{cc} \; & P_{\tilde{x}\tilde{z}}=\sum_{i=0}^{2n}w_{i}^{c}{\tilde{x}}_{i}^{\zeta }\left(t_{k}^{e}|t_{k-1}^{e}\right){\left({\tilde{z}}_{i}\left(t_{k}^{e}|t_{k-1}^{e}\right)\right)}^{\prime } \end{array}$$

(61) $$\begin{array}{cc} \; & P_{\tilde{z}}=\sum_{i=0}^{2n}w_{i}^{c}{\tilde{z}}_{i}\left(t_{k}^{S}|t_{k-1}^{S}\right){\left({\tilde{z}}_{i}\left(t_{k}^{S}|t_{k-1}^{S}\right)\right)}^{\prime }+R\left(t_{k}^{S}\right) \end{array}$$

(62) $$\begin{array}{cc} \; & K_{k}=P_{\tilde{x}\tilde{z}}P_{\tilde{z}}^{-1} \end{array}$$

(63) $$\begin{array}{cc} \; & \hat{x}(t_{k}^{e})=\hat{x}(t_{k}^{e}|t_{k-1}^{e})+K_{k}\left(z\left(t_{k}^{S}\right)-\hat{z}(t_{k}^{S}|t_{k-1}^{S})\right) \end{array}$$

(64) $$\begin{array}{cc} \; & P\left(t_{k}^{e}\right)=P(t_{k}^{e}|t_{k-1}^{e})-K_{k}P_{\tilde{z}}{\left(K_{k}\right)}^{\prime }. \end{array}$$

where $R(t_{k}^{S})$ denotes the variance of measurement noise at the sampling instant $t_{k}^{S}$.

4. The iterated stage

On the Basis of the UKF algorithm, an iteration method in [21] is adopted to estimate the target state and covariance matrix.

(i) Set the initial value $\left[\hat{x}(t_{k}^{e}\right){]}^{0}=\hat{x}(t_{k}^{e}|t_{k-1}^{e})$, ${\left[P\left(t_{k}^{e}\right)\right]}^{0}=P(t_{k}^{e}\left|t_{k-1}^{e}\right)$ and $\left[\hat{x}(t_{k}^{e}\right){]}^{1}=\hat{x}(t_{k}^{e})$,
  ${\left[P\left(t_{k}^{e}\right)\right]}^{1}=P\left(t_{k}^{e}\right)$. Let g=1,j=2 (*j* represents the jth iterate)
(ii) Generate a new sigma point for iterated state
  (65) $${\left[\xi \left(t_{k}^{e}\right)\right]}^{j}=\left[{\left[\hat{x}\left(t_{k}^{e}\right)\right]}^{j-1}\;\;\;\;\;\;{\left[\hat{x}\left(t_{k}^{e}\right)\right]}^{j-1}+\sqrt{\left(n+\lambda \right){\left[P\left(t_{k}^{e}\right)\right]}^{j-1}}\;\;\;\;\;\;{\left[\hat{x}\left(t_{k}^{e}\right)\right]}^{j-1}-\sqrt{\left(n+\lambda \right){\left[P\left(t_{k}^{e}\right)\right]}^{j-1}}\right],$$
(iii) Repeat the time update and measurement update procedure (47)–(64) as follows
  (66) $$\begin{array}{cc} {\left[\xi_{i}^{-}\left(t_{k}^{e}\right)\right]}^{j,\;l} & =\left[{\left[\xi_{i}^{-}\left(t_{k}^{e}\right)\right]}^{j,\;l-1}-{\left(J_{s}^{T}J_{s}\right)}^{-1}J_{s}^{T}s\left({\left[\xi_{i}^{-}\left(t_{k}^{e}\right)\right]}^{j,\;l-1},{\left[\xi_{i}\left(t_{k}^{e}\right)\right]}^{j}\right)\right] \end{array}$$
  (67) $$\begin{array}{cc} \; & \left[{\hat{x}}^{-}(t_{k}^{e}\right){]}^{j}=\sum_{i=0}^{2n}w_{i}^{m}\left[{\hat{\xi }}_{i}^{-}(t_{k}^{e}\right){]}^{j} \end{array}$$
  (68) $$\begin{array}{cc} \;\; & {\left[{\hat{z}}_{i}^{-}\left(t_{k}^{S}\right)\right]}^{j}=h\left({\left[\xi_{i}\left(t_{k}^{e}\right)\right]}^{j}\right) \end{array}$$
  (69) $$\begin{array}{cc} \; & {\left[{\hat{z}}^{-}\left(t_{k}^{S}\right)\right]}^{j}=\sum_{i=0}^{2n}w_{i}^{m}\;{\left[{\hat{z}}_{i}\left(t_{k}^{S}\right)\right]}^{j} \end{array}$$
  (70) $$\begin{array}{cc} \; & {\left[{\tilde{x}}_{i}^{\zeta }\left(t_{k}^{e}\right)\right]}^{j}=\left[{\hat{\zeta }}_{i}^{-}(t_{k}^{e}\right){{]}^{j}-\left[{\hat{x}}^{-}(t_{k}^{e}\right)]}^{j} \end{array}$$
  (71) $$\begin{array}{cc} \; & {\left[{\tilde{z}}_{i}\left(t_{k}^{S}\right)\right]}^{j}=\left[{\hat{z}}_{i}^{-}(t_{k}^{S}\right){{]}^{j}-\left[{\hat{z}}^{-}(t_{k}^{S}\right)]}^{j} \end{array}$$
  (72) $$\begin{array}{cc} \; & P_{\tilde{x}\tilde{z}}^{j}=\sum_{i=0}^{2n}w_{i}^{c}{\left[{\tilde{x}}_{i}^{\zeta }\left(t_{k}^{e}\right)\right]}^{j}{\left({\left[{\tilde{z}}_{i}\left(t_{k}^{e}\right)\right]}^{j}\right)}^{\prime } \end{array}$$
  (73) $$\begin{array}{cc} \; & P_{\tilde{z}}^{j}=\sum_{i=0}^{2n}w_{i}^{c}{\left[{\tilde{z}}_{i}\left(t_{k}^{S}\right)\right]}^{j}{\left({\left[{\tilde{z}}_{i}\left(t_{k}^{S}\right)\right]}^{j}\right)}^{\prime }+R\left(t_{k}^{S}\right) \end{array}$$
  (74) $$\begin{array}{cc} \; & K_{k}^{j}=P_{\tilde{x}\tilde{z}}^{j}{\left(P_{\tilde{z}}^{j}\right)}^{-1} \end{array}$$
  (75) $$\begin{array}{cc} \; & \left[\hat{x}(t_{k}^{e}\right){]}^{j}={\left[{\hat{x}}^{-}(t_{k}^{e})\right]}^{j}+K_{k}^{j}\left(z\left(t_{k}^{S}\right)-{\left[{\hat{z}}^{-}(t_{k}^{S})\right]}^{j}\right) \end{array}$$
  (76) $$\begin{array}{cc} \; & {\left[P\left(t_{k}^{e}\right)\right]}^{j}={\left[P(t_{k}^{e})\right]}^{j-1}-K_{k}^{j}P_{\tilde{z}}^{j}{\left(K_{k}^{j}\right)}^{\prime }. \end{array}$$
(iv) Define the judgment criteria equations
  (77) $$\begin{array}{cc} \; & {\left[\hat{z}\left(t_{k}^{S}\right)\right]}^{j}=h{\left(\left[\hat{x}(t_{k}^{e}\right)\right]}^{j}) \end{array}$$
  (78) $$\begin{array}{cc} \; & {\left[\tilde{x}\left(t_{k}^{e}\right)\right]}^{j}=\left[\hat{x}(t_{k}^{e}\right){{]}^{j}-\left[\hat{x}(t_{k}^{e}\right)]}^{j-1} \end{array}$$
  (79) $$\begin{array}{cc} \; & {\left[\tilde{z}\left(t_{k}^{S}\right)\right]}^{j}=z\left(t_{k}^{S}\right)-{\left[\hat{z}\left(t_{k}^{S}\right)\right]}^{j}. \end{array}$$
(v) The termination criteria for iterative process:
  If the following inequality satisfies
  (80) $${\left({\left[\tilde{x}\left(t_{k}^{e}\right)\right]}^{j}\right)}^{\prime }{\left[P^{-1}(t_{k}^{e})\right]}^{j-1}{\left[\tilde{x}\left(t_{k}^{e}\right)\right]}^{j}+{\left({\left[\tilde{z}\left(t_{k}^{S}\right)\right]}^{j}\right)}^{\prime }R^{-1}\left(t_{k}^{S}\right){\left[\tilde{z}\left(t_{k}^{S}\right)\right]}^{j}\geq {\left({\left[\tilde{z}\left(t_{k}^{S}\right)\right]}^{j-1}\right)}^{\prime }R^{-1}\left(t_{k}^{S}\right){\left[\tilde{z}\left(t_{k}^{S}\right)\right]}^{j-1}.$$
  or if *j* reaches the $N_{max}$, stop the iterative and output $\hat{x}(t_{k}^{e}{)=\left[\hat{x}(t_{k}^{e}\right)]}^{j},P\left(t_{k}^{e}\right)={\left[P\left(t_{k}^{e}\right)\right]}^{j}$. Otherwise, let g=η·g,j=j+1 and go back to step (iii).

Different from the standard Gauss–Helmert Unscented Kalman Filter (GH-UKF) method, GH-IUKF corrects the measurement to adjust the estimation of state and adaptively approximate the true value. The estimation error of state is expected lower than GH-UKF after the iteration is terminated. In addition, GH-IUKF can respond to the iterated measurements rapidly, and keep a faster convergence speed even if the initial error is large. Algorithm 1 summarizes the main process of the GH-IUKF algorithm.

**Algorithm 1** The GH-IUKF algorithm.

**First stage:**
(1) Calculate 2n+1 sigma points $\xi^{i}\left(t_{k-1}^{e}\right)$ for given $\left(x\left(t_{k-1}^{e}\right),P\left(t_{k-1}^{e}\right)\right)$ by (43).(2) Calculate the Jacobian matrix $J_{s}$ by Equation (48) and predicted sigma point $\xi^{i}\left(t_{k}^{e}|t_{k-1}^{e}\right)$ by the implicit stae transmission function s(·).l = 1, I = 1;**while** I = 1 **do** ${\left[\xi^{i}\left(t_{k}^{e}|t_{k-1}^{e}\right)\right]}^{l}={\left[\xi^{i}\left(t_{k}^{e}|t_{k-1}^{e}\right)\right]}^{l-1}-{\left(J_{s}^{T}J_{s}\right)}^{-1}J_{s}^{T}s\left[{\left[\xi^{i}\left(t_{k}^{e}|t_{k-1}^{e}\right)\right]}^{l-1},\xi^{i}\left(t_{k-1}^{e}\right)\right],l=l+1$ **if**$\left|\frac{SSR^{l}-SSR^{l-1}}{SSR^{l}}\right|<\sigma$ or $l>L_{\max}$ **then**  $\xi^{i}\left(t_{k}^{e}\left|t_{k-1}^{e}\right.\right)={\left[\xi^{i}\left(t_{k}^{e}\left|t_{k-1}^{e}\right.\right)\right]}^{l}$, I=0 **end if**
**end while**
(3) Update the first stage state $\hat{x}\left(t_{k}^{e}\right)$ and error covariance $P(t_{k}^{e})$ by Equations (52) to (64).
**Iterated stage:**
(1) Let the initial value $\left[\hat{x}(t_{k}^{e}\right){]}^{1}=\hat{x}(t_{k}^{e})$, ${\left[P\left(t_{k}^{e}\right)\right]}^{1}=P\left(t_{k}^{e}\right)$.(2) Iterate the state and error covariance until they are satisfied with the termination criteriaj = 2, g = 1;**for** j:$N_{max}$ **do** Execute the procedure (65)–(76) **if** ${\left({\left[\tilde{x}\left(t_{k}^{e}\right)\right]}^{j}\right)}^{\prime }{\left[P^{-1}(t_{k}^{e})\right]}^{j-1}{\left[\tilde{x}\left(t_{k}^{e}\right)\right]}^{j}+{\left({\left[\tilde{z}\left(t_{k}^{S}\right)\right]}^{j}\right)}^{\prime }R^{-1}\left(t_{k}^{S}\right){\left[\tilde{z}\left(t_{k}^{S}\right)\right]}^{j}\geq {\left({\left[\tilde{z}\left(t_{k}^{S}\right)\right]}^{j-1}\right)}^{\prime }R^{-1}\left(t_{k}^{S}\right){\left[\tilde{z}\left(t_{k}^{S}\right)\right]}^{j-1}$ **then**  Break **else**  g=ηg,j=j+1 **end if**
**end for**

$\hat{x}(t_{k}^{e}{)=\left[\hat{x}(t_{k}^{e}\right)]}^{j},P\left(t_{k}^{e}\right)={\left[P\left(t_{k}^{e}\right)\right]}^{j}$



## 5. Numerical Simulation

This section considers a maneuvering observer (equipped with three sensors, which is applied to obtain the bearing angle, elevation angle and Doppler frequency, respectively) to track an underwater target in three-dimensional space. The underwater target scenarios are modified according to the test case in [3,34]. The initial position of single maneuvering observer $\left(x_{0}^{S},y_{0}^{S},z_{0}^{S}\right)$ is (−500,0,0) m and its’ trajectory is selected to move on a curve in the horizontal plane, where the velocity in the *x*-axis direction maintains at ${\dot{x}}^{S}\left(t_{k}^{S}\right)$ = 12 m/s. Then the relationships of observer motion parameters are defined as the following equations

(81) $$x^{S}=x_{0}^{S}+{\dot{x}}^{S}t_{k}^{S}$$

(82) $$y^{S}=y_{0}^{S}+200\sin\left(\left(-2\pi /1000\right)x^{S}\right)$$

(83) $${\dot{y}}^{S}=-\frac{2\pi }{5}\cos\left(\left(-2\pi /1000\right)x^{S}\right){\dot{x}}^{S}$$

(84) $$z^{S}=z_{0}^{S},\;\;\;{\dot{z}}^{S}=0.$$

In this scenario, we assume a single target moves forward with the constant velocity (−20,10,0) m/s, and its’ initial location is (2000,1200,200) m. The underwater sound propagation speed *c* is 1480 m/s, the initial source frequency from the target is $f_{0}$ = 365 Hz, and the initial signal emission time $t_{0}^{e}$ is calculated according to (19). Both of the bearing angle and elevation angle measurement noise standard deviation are $\sigma_{\theta }^{v}=\sigma_{\varphi }^{v}$ = 3°, Doppler frequency measurement error standard deviation is $\sigma_{f}^{v}$ = 5 Hz. The sensors sampling interval is *T* = 1 s, and the observer obtains measurement data from $t_{s}$ = 6 s to $t_{s}$ = 105 s. The system process noise covariance matrix is given by

(85) $$q=diag[{\left(0.1\;m/s^{2}\right)}^{2}\;{\left(0.1\;m/s^{2}\right)}^{2}\;{\left(0.1\;m/s^{2}\right)}^{2}\;{\left(0.01\mathrm{Hz}\right)}^{2}\;{\left(0.01\;s\right)}^{2}],$$

The initial state $x_{0}^{*}$ is randomly generated by the true value and the initial error covariance according to [30]

(86) $$P\left(t_{0}^{e}\right)=diag\left[{\left(100\;m\right)}^{2}\;{\left(100\;m\right)}^{2}\;{\left(100\;m\right)}^{2}\;{\left(3\;m/s\right)}^{2}\;{\left(3\;m/s\right)}^{2}\;{\left(3\;m/s\right)}^{2}\;{\left(1\mathrm{Hz}\right)}^{2}\;{\left(0.1\;s\right)}^{2}\right].$$

The position root mean square error (RMSE) is given by

(87) $$RMSE\left({\hat{t}}_{k}^{e}\right)=\sqrt{\frac{1}{N}\sum_{i=1}^{N}\left[{\left(\hat{x}\left({\hat{t}}_{k}^{e}\right)-x\left({\hat{t}}_{k}^{e}\right)\right)}^{2}+{\left(\hat{y}\left({\hat{t}}_{k}^{e}\right)-y\left({\hat{t}}_{k}^{e}\right)\right)}^{2}+{\left(\hat{z}\left({\hat{t}}_{k}^{e}\right)-z\left({\hat{t}}_{k}^{e}\right)\right)}^{2}\right]}.$$

where *N* is the numbers of Monte-Carlo simulation, $\hat{x}\left({\hat{t}}_{k}^{e}\right)$, $\hat{y}\left({\hat{t}}_{k}^{e}\right)$ and $\hat{z}\left({\hat{t}}_{k}^{e}\right)$ are the estimation of position, $x({\hat{t}}_{k}^{e})$, $y({\hat{t}}_{k}^{e})$ and $z({\hat{t}}_{k}^{e})$ are the true target position.

The statistical test for filter consistency based on the average normalized estimation error squared (ANEES) which is given by [39]

(88) $$\varepsilon \left({\hat{t}}_{k}^{e}\right)=\frac{1}{nN}\sum_{i=1}^{N}\left(x_{i}({\hat{t}}_{k}^{e}\right)-{\hat{x}}_{i}\left({\hat{t}}_{k}^{e}\right){)}^{T}{\left[P\left({\hat{t}}_{k}^{e}\right)\right]}^{-1}\left(x_{i}({\hat{t}}_{k}^{e}\right)-{\hat{x}}_{i}\left({\hat{t}}_{k}^{e}\right))$$

where *n* is the dimension of the state vector, $\left(x_{i}({\hat{t}}_{k}^{e}\right)-{\hat{x}}_{i}\left({\hat{t}}_{k}^{e}\right))$ and $P({\hat{t}}_{k}^{e})$ are the filter estimation error and covariance for the *i*th Monte-Carlo simulation at the instant ${\hat{t}}_{k}^{e}$. The acceptance interval threshold $\sigma_{1}$ and $\sigma_{2}$ at level α=0.05 are calculated by [40]

(89) $$\sigma_{1,2}\approx \frac{1}{2nN}{\left(\pm 1.96+\sqrt{2nN-1}\right)}^{2}$$

Three extra algorithms are tested to compare with the proposed algorithm in this paper.

- GH-IUKF: The proposed algorithm in this paper. It considers the existence of signal time delay between the target and the observer, using the estimated state and its covariance matrix to perform the second-stage iterative process. It extends the emission times $t_{k}^{e}$ to the state by GHM in both of the two stages of filtering. The iterate number is set to $N_{max}=3$.
- GH-UKF: This is the existing approach which uses the GHM framework, and it expands $t_{k}^{e}$ as a variable into the state.
- IUKF-D: This method only takes $t_{k}^{d}$ as an unknown parameter in the first stage, while it will become a certain parameter in the iterated stage. The iterate number is set to $N_{max}=3$.
- UKF-D: The state transition model of this method is based on the GMM framework, and the signal propagation delay $t_{k}^{d}$ is treated as an unknown parameter, which is obtained by an iterative method while the state is updated.

**Figure 4:**
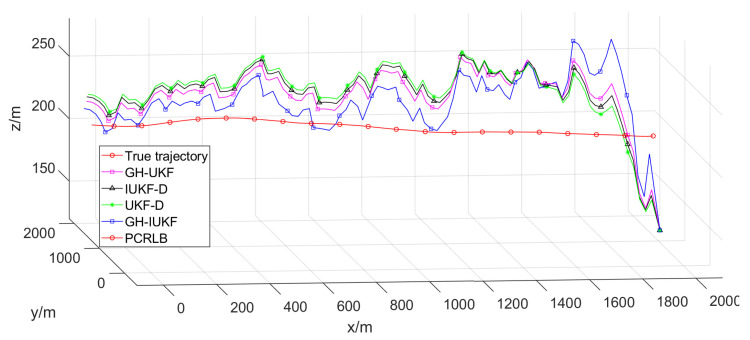
Tracking trajectory in 3D space.

Figure 4 depicts the target trajectory without noise and the estimated trajectory in 3-D space. The observer maneuvers forward on the surface of water, while the target keeps moving underwater with a constant speed at a certain depth. It can be seen that all approaches can effectively track the target during the entire tracking process. Where GH-IUKF is superior to other approaches in both accuracy and convergence speed, the performance of UKF-D is the worst of all, and it is not sensitive to the measurement update and the tracking trajectory will always be unable to get close to the true target trajectory.

**Figure 5:**
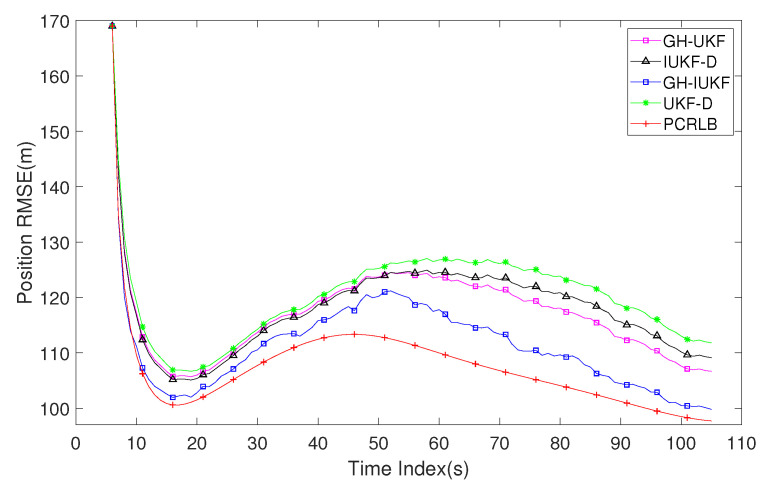
RMSE of the estimated position versus time index k.

Figure 5 shows the position RMSE of the target with the above four algorithms under 1000 Monte Carlo runs. In order to compare the performance of all algorithms more clearly, the posterior Cramer–Rao lower bound (PCRLB) in [41] is selected as the performance criteria for comparison (the signal emission time is assumed to be known while calculating the bound). The observer starts to receive measurements at $t_{s}$ = 6 s and end at $t_{s}$ = 105 s with a total number of 100 data for all methods. It can be seen that GH-IUKF shows the highest accuracy of estimation among the four algorithms, and it is very close to PCRLB. This is because the algorithm adopts two stages filtering, which can adjust the state estimate and adaptively decrease the errors. Furthermore, the algorithm applies the GHM state transition model, which is superior to GMM. The signal emission time is expanded as a state variable and the additional information can provide better performance. Correspondingly, the overall trend of GH-UKF and GH-IUKF are basically the same. The RMSE of these two algorithms has a significant drop when $t_{s}$ = 50 s, and the convergence speed is faster than the other two algorithms. However, its accuracy is not as good as GH-IUKF and cannot be closer to PCRLB. The tracking performance of IUKF-D is slightly better than GH-UKF before $t_{s}$ = 50 s. However, due to the disadvantage of the model, it was overtaken by the latter in the second half of the tracking process.

**Figure 6:**
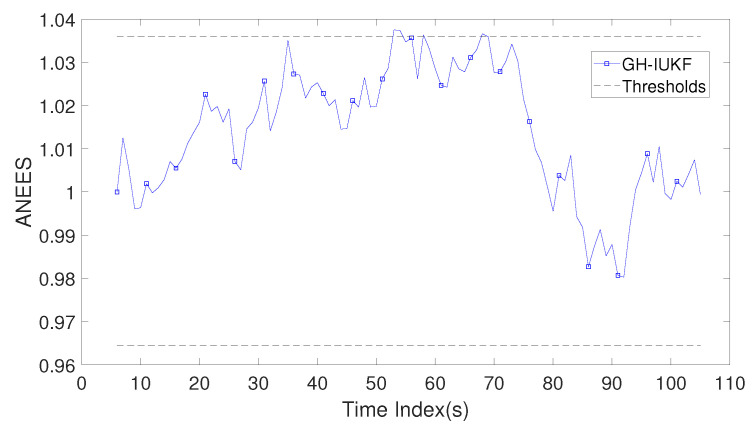
Average normalized estimation error squared (ANEES) of the Gauss–Helmert Iterated Unscented Kalman Filter (GH-IUKF) versus time index k.

Figure 6 depicts the result of ANEES for the proposed method in this paper. The acceptance threshold $\sigma_{1}$ = 1.0360 and $\sigma_{2}$ = 0.9645. The ANEES in Figure 6 is close to 1, and the results for the majority of the time are within the confidence interval, which means the estimation error and the covariance are compatible with each other, and the estimation of the filter is reliable and credible.

As the initial error covariance of the filter is relatively large, the RMSE will change rapidly at the beginning from $t_{s}$ = 6 s to $t_{s}$ = 9 s, and the difference of estimation performance between different algorithms is very small. In order to make the curves in the graph easier to distinguish, the horizontal axis of the following graphs related to RMSE will start from $t_{s}$ = 10 s.

**Figure 7:**
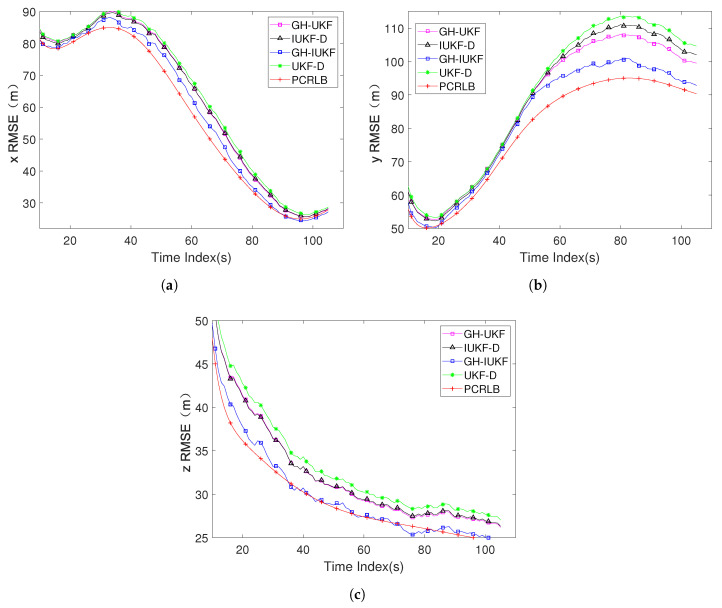
The RMSE components of target position and velocity: (**a**) RMSE of the position in *x* axis; (**b**) RMSE of the position in *y* axis; (**c**) RMSE of the position in *z* axis.

Figure 7 shows the RMSE components (position and velocity) with all stated algorithms. The target is moving close to the observer on the *x*-axis, which will cause the increment in varying rates of the measurement angle. Therefore, in Figure 6a, the performance of all algorithms is stable, and eventually converges to PCRLB; comparatively, the target is far away from the observation station on the *y*-axis. As shown in Figure 6b, the tracking performance of the entire system decreases, which has a great impact on all algorithms. Only GH-IUKF can resist this negative effect and the RMSE eventually approaches PCRLB. Both the target and the observer are kept at a certain depth. In Figure 6c, the system performance on the *z*-axis is very stable, and all algorithms converge quickly.

**Figure 8:**
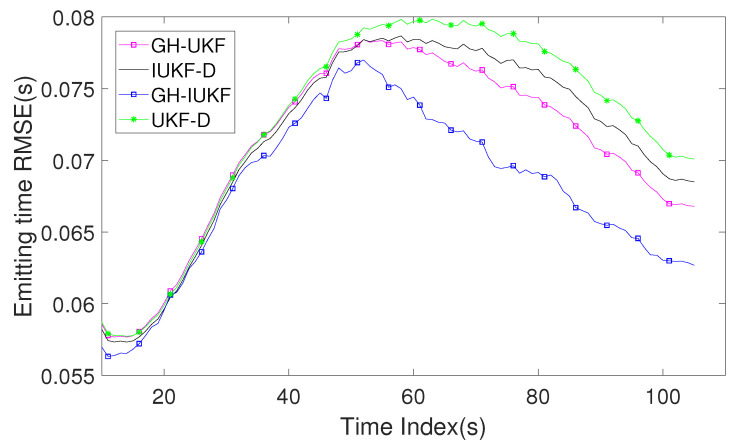
RMSE of the estimated signal emission time versus time index k.

Figure 8 describes the RMSE of the estimated signal emission time. GH-IUKF and GH-UKF regard the emission times as a state variable, while IUKF-D and UKF-D calculate the emission time by (19) after the target state is estimated because these latter two methods do not expand the unknown signal emission time to the state.

**Figure 9:**
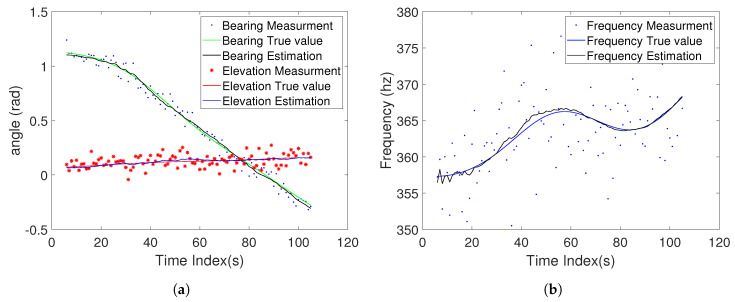
(**a**) The true angle, the angle measurement and estimated angle for GH-IUKF; (**b**) the true frequency, the frequency measurement and estimated frequency for GH-IUKF.

Figure 9 depicts the angle and frequency calculated based on GH-IUKF estimate and it is compared with the measurement and true value. By the influence of noise, the measurements will fluctuate widely compared with the true value. The measurement estimation calculated based on the estimated state value by the GH-IUKF algorithm filter proposed in this paper is very close to the real measurements, which further proves the high accuracy of the GH-IUKF algorithm. Table 1 gives a performance comparison for the above methods, which includes time costs for each simulation and average RMSE for 1000 Monte-Carlo runs with different angle measurement noise standard deviations.

**Figure 10:**
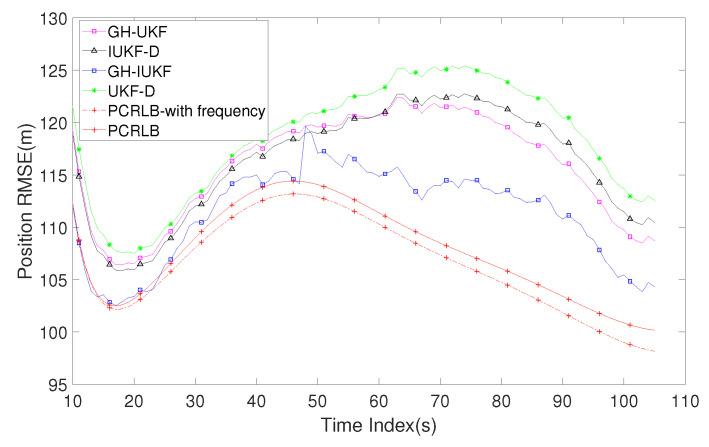
RMSE of the estimated position without Doppler frequency information.

Figure 10 depicts the position of the RMSE of the target without Doppler frequency. The PCRLB-with frequency in this figure is to compare the effect of the Doppler frequency measurement information for the filter on the overall estimation performance. Compared with Figure 5, we found that the additional Doppler frequency measurement information has slightly improved the accuracy of all methods. The underwater acoustic propagation speed is approximately five times that in air, but the velocity of the underwater target is much slower than the ground or air target, so the Doppler measurement has limited improvement for the underwater target tracking accuracy. However, by comparing the two PCRLBs in Figure 9, we found that the Doppler measurement can still improve the overall performance of the system and make the system more stable.

## 6. Conclusions

Acoustic signal propagation delay cannot be ignored in underwater target tracking problems, and it varies with time, which has a continuous negative impact on the tracking effect. In this paper, GHM is introduced to expand the unknown signal transmission time as an unknown variable to the state, and the solution is based on the optimization method. On the basis, this paper further utilizes the estimated state and covariance for the second stage iteration to improve the algorithm accuracy. In addition, we also apply Doppler frequency measurement to improve the overall performance of the system and expand it into a three-dimensional space. The simulation shows that the proposed method GH-IUKF in this paper equips higher accuracy and more stable performance in underwater environments than other algorithms. Aiming at the complex underwater environmental characteristics, future research will focus on the initial value calculation method of target tracking based on underwater signal propagation delay.

## Figures and Tables

**Table 1:** Performance comparison.

	GH-IUKF	GH-UKF	IUKF-D	UKF-D
Time per Run (ms)	134	83	50	24
1° Average RMSE (m)	77.04	78.96	79.71	82.19
2° Average RMSE (m)	97.15	100.38	101.43	105.61
3° Average RMSE (m)	111.50	117.22	118.09	120.30
5° Average RMSE (m)	133.15	139.96	140.44	151.75

## Appendix A

The judgment of the singularity for Jacobian matrix $J_{s}$ is

(A1) $$\begin{array}{cc} \left|J_{s}\right| & =[1+{\dot{x}}^{T}\left(t_{k-1}^{e}\right)\frac{x^{T}-x^{S}}{dc}+{\dot{y}}^{T}\left(t_{k-1}^{e}\right)\frac{y^{T}-y^{S}}{dc}+{\dot{z}}^{T}\left(t_{k-1}^{e}\right)\frac{z^{T}-z^{S}}{dc}]\\ \; & =[1+\frac{\dot{x}\left(t_{k-1}^{e}\right)}{c}\left(\sin\phi \sin\theta \cos\psi \cos\varphi +\cos\phi \cos\theta \cos\psi \cos\varphi +\sin\psi \sin\varphi \right)]. \end{array}$$

where $\phi \in [-\frac{\pi }{2},\frac{\pi }{2}]$ is the angle between the target moving direction and *y*-axis in the horizontal plane; $\psi \in [-\frac{\pi }{2},\frac{\pi }{2}]$ is the angle between the target moving direction and horizontal plane.

Define the parameter μ as the follow equation

(A2) μ=sinϕsinθcosψcosφ+cosϕcosθcosψcosφ+sinψsinφ,

1. When θ=ϕ,φ=ψ
  (A3) $$\mu =\sin^{2}\theta \cos^{2}\varphi +\cos^{2}\theta \cos^{2}\varphi +\sin^{2}\varphi =1,$$
  $\det(J_{s})$ will be zero if the target velocity $\dot{x}\left(t_{k-1}^{e}\right)=-c$. This situation corresponds to the target moving towards or away from the observer with the speed −c.
2. When θ=−ϕ
  (A4) $$\mu =\cos^{2}\varphi (\cos^{2}\theta -\sin^{2}\theta )\pm \sin^{2}\varphi .$$
  $\det(J_{s})$ will be zero if any of the following terms are held
  (i) $\theta =\frac{\pi }{2},\;\phi =-\frac{\pi }{2}$ or $\theta =-\frac{\pi }{2},\;\phi =\frac{\pi }{2}$ and φ=−ψ, $\dot{x}\left(t_{k-1}^{e}\right)=c$
  (ii) $\varphi =\psi =\pm \frac{\pi }{2}$ and $\dot{x}\left(t_{k-1}^{e}\right)=-c$

Except for the above special cases, the sigma points in (47) and (66) will always converge on the condition that the initial value is selected reasonably.
